# Uncommon Manifestations of Newly Diagnosed B-cell Chronic Lymphocytic Leukemia Prompting the Concurrent Diagnosis of Advanced Prostate Cancer: A Case Report

**DOI:** 10.7759/cureus.90223

**Published:** 2025-08-16

**Authors:** Christos Stafylidis, Iraklis Patsialos, Dimitra Stefanou, Eleftheria Lakiotaki, Panagiotis Diamantopoulos

**Affiliations:** 1 Hematology Unit, First Department of Internal Medicine, National and Kapodistrian University of Athens, Laikon General Hospital, Athens, GRC; 2 First Department of Pathology, National and Kapodistrian University of Athens, Laikon General Hospital, Athens, GRC

**Keywords:** chronic lymphocytic leukemia (cll), hematologic malignancies, prostate cancer, secondary malignancies, solid tumors

## Abstract

Patients with chronic lymphocytic leukemia (CLL) have an increased risk for developing secondary malignancies (SMs), which usually follow an aggressive clinical course and are associated with unfavorable survival rates. Herein, we describe a patient diagnosed with CLL, who also presented with uncommon findings such as extremely elevated alkaline phosphatase levels, abnormal coagulation studies, and leucoerythroblastic reaction, that ultimately resulted in the diagnosis of concurrent metastatic prostate cancer. Early recognition of findings indicative of SM in CLL patients, even during early stages, is crucial, and physicians should stay alert. Furthermore, CLL patients should undergo more frequent screening tests for cancer as compared to the general population.

## Introduction

Chronic lymphocytic leukemia (CLL) represents the most common leukemia in adults and is characterized by the presence of a monoclonal population of mature B-cells in the bone marrow (BM) and the peripheral blood, whereas lymph nodes and spleen are also frequently involved [1]. At diagnosis, most patients are usually asymptomatic; however, palpable lymphadenopathy, splenomegaly, and/or B symptoms may be detected [1]. Inherent immunodeficiency and increased susceptibility to infections, due to defects of the innate and adaptive immune systems, along with hypogammaglobulinemia, constitute another hallmark of the disease [1]. CLL has also been associated with autoimmune manifestations, such as autoimmune hemolytic anemia [1]. Importantly, an increased incidence of secondary malignancies (SMs) has been reported among patients with CLL, including solid organ tumors (SOTs), such as skin, prostate, breast, gastrointestinal, and lung cancers, as well as hematological malignancies (HMs) [1-3]. Prolonged survival and long-term immunosuppression, which are both disease-associated and treatment-related, may account for this elevated risk for SMs in CLL patients [1-3]. Although SMs’ presenting features and clinical course in this patient population are not well-defined, previous reports have demonstrated an increased occurrence of SMs in older patients and advanced CLL stage or in those who had received prior CLL treatments and a more aggressive clinical course, which is correlated with inferior survival [1, 3, 4]. Herein, we report a rare case of a patient with newly diagnosed CLL who also presented with uncommon findings that eventually led to the concurrent diagnosis of stage IV prostate cancer.

## Case presentation

A 78-year-old man presented to the emergency department with shortness of breath and hypoxemia. The patient was a nonsmoker and had a medical history of atrial fibrillation and right heart failure under treatment with apixaban, bisoprolol, and furosemide.

General clinical examination was unremarkable except for edema of the extremities. No hepatomegaly, splenomegaly, or lymphadenopathy was observed. The patient was hospitalized and immediately started on intravenous diuretics and supplemental oxygen therapy. A computed tomography pulmonary angiogram was negative for pulmonary embolism.

The initial laboratory evaluation, as shown in Table 1, revealed a markedly elevated white blood cell count with prominent lymphocytosis, normocytic anemia, and thrombocytopenia along with significantly elevated levels of alkaline phosphatase (ALP), lactate dehydrogenase (LDH), and uric acid. Serum glutamic pyruvic and oxaloacetic transaminases (SGPT and SGOT, respectively) and γ-glutamethyltransferase (γGT) were slightly elevated while total bilirubin and serum albumin were within normal limits. Kidney function tests and electrolytes were within normal range except for low calcium levels. His β2-microglobulin levels were slightly elevated. Low haptoglobin levels were also found with a negative direct antiglobulin test, whereas vitamin B12 and folic acid levels were also within normal range. Hemostasis screening tests indicated prolonged prothrombin and activated partial thromboplastin time, elevated d-dimers, and low fibrinogen levels. Remarkably, the patient had a normal complete blood count and blood chemistry tests eight months ago. Additionally, the patient’s serology tests were negative for HAV, HBV, HCV, HIV, cytomegalovirus (CMV), and Epstein-Barr virus (EBV), and he also underwent an upper abdominal ultrasound, which yielded no findings.

**Table 1:** Main laboratory findings of the patient WBC: white blood cell; Hct: hematocrit; Hb: hemoglobin; PLT: platelet; PT: prothrombin time; aPTT: activated partial thromboplastin time; DAT: direct antiglobulin test; SGOT: serum glutamic-oxaloacetic transaminase; SGPT: serum glutamic pyruvic transaminase; ALP: alkaline phosphatase; γGT: gamma-glutamyl transferase; CRP: C-reactive protein

Laboratory Test	Result	Range
WBC count	83.47 × 10^9^/L	4.5-11 × 10^9^/L
Neutrophil count	2.5 × 10^9^/L	1.5-6.6 × 10^9^/L
Lymphocyte count	81.13 × 10^9^/L	1.2-3.4 × 10^9^/L
Hct	27.20%	40%-54%
Hb	8.5 g/dL	13.5-18 g/dL
MCV	93.2 fL	80-96 fL
Corrected reticulocyte count	1.79%	
PLT count	66 x 10^9^/L	140-440 x 10^9^/L
PT	17.9 sec	11-13 sec
INR	2.6	0.90-1.20
aPTT	48 sec	29-40 sec
Fibrinogen	180 mg/dL	180-400 mg/dL
d-dimers	6.9 μg/mL	0.20-0.50 μg/mL
Haptoglobin	27 mg/dL	>50 mg/dL
DAT	Negative	
Urea	65 mg/dL	15-43 mg/dL
Creatinine	1.2 mg/dL	0.67-1.17 mg/dL
Uric acid	10.1 mg/dL	3.4-7 mg/dL
K^+^	4.1 mmol/L	3.7-4.9 mmol/L
Na^+^	136 mmol/L	136-143 mmol/L
Ca^2+^	7.9 mg/dL	8.6-10.2 mg/dL
PO_4_^3-^	3.3 mg/dL	2.5-4.5 mg/dL
LDH	1800 U/L	135-225 U/L
SGOT	58 U/L	15-40 U/L
SGPT	77 U/L	<41 U/L
ALP	11.234 U/L	40-129 U/L
γGT	70 U/L	8-61 U/L
Total bilirubin	1.13 mg/dL	0.30-1.20 mg/dL
Direct bilirubin	0.5 mg/dL	0.00-0.30 mg/dL
CRP	15 mg/L	0-5 mg/L
Β2-microglobulin	3.01 mg/L	0.8-2.2 mg/L

**Figure 1:**
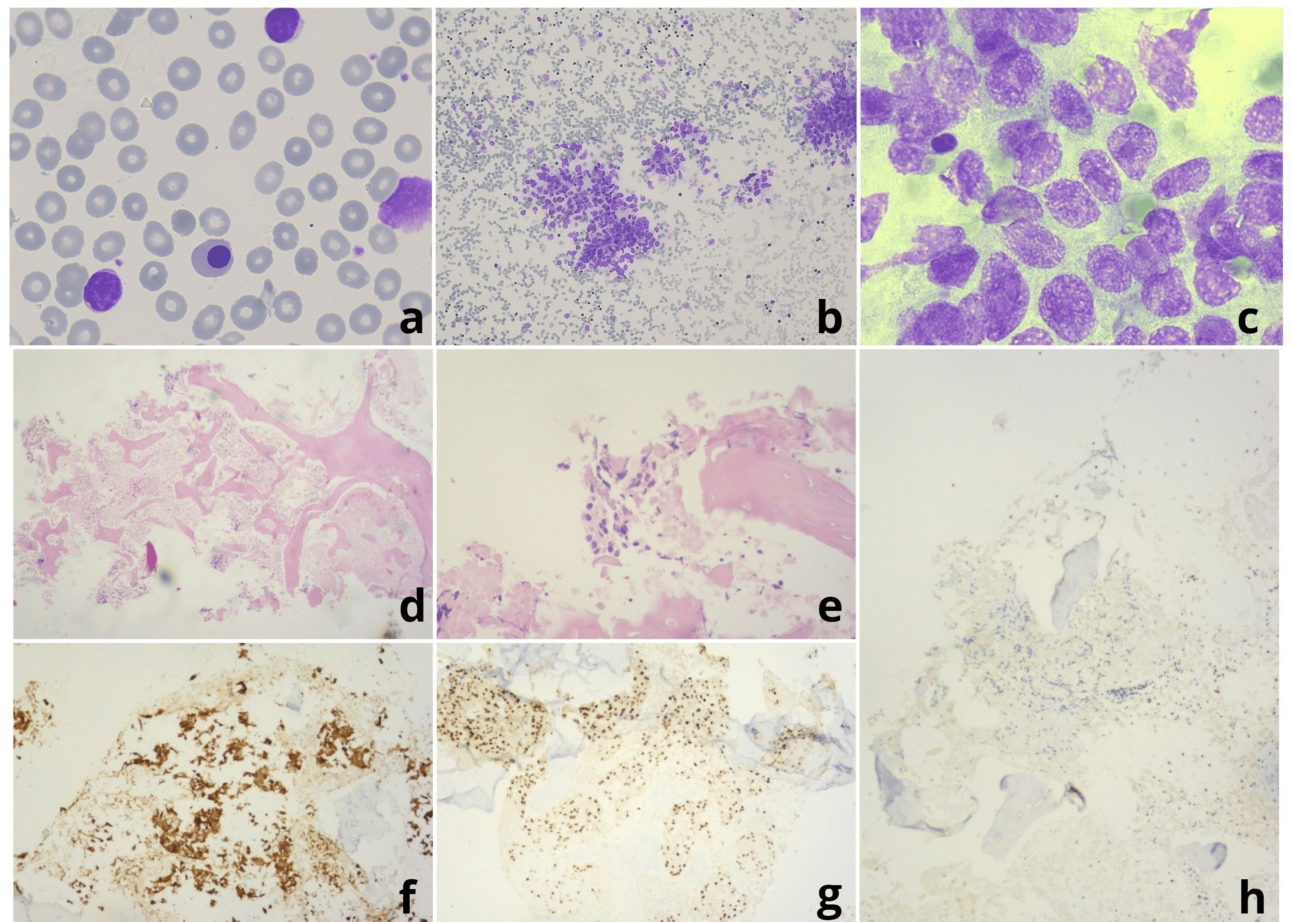
Peripheral blood smear, bone marrow aspirate, and trephine biopsy findings a) Peripheral blood smear (Wright-Giemsa stain, ×100) revealing the presence of mature-appearing lymphocytes along with a smudge cell and a nucleated red blood cell. b-c) Bone marrow (BM) aspiration showing clusters of non-hematopoietic cells. Wright-Giemsa stain, 10× (b) and 100× (c). d-h) BM trephine biopsy images. Hematoxylin and eosin stain, 40× (d) and 200× (e). BM architecture is effaced, and large necrotic areas are evident. Sheets of atypical neoplastic cells are seen infiltrating the BM (e). f) CKAE1/AE3 immunostain (100×). The neoplastic population is of epithelial origin, as pankeratin stain is positive on neoplastic cells and necrotic areas. g) NKX3.1 immunostain (100×). Nuclear staining on neoplastic cells is compatible with prostatic origin. h) CD23 immunostain (100×). Surprisingly, no chronic lymphocytic leukemia cells are detected.

Examination of a peripheral blood smear showed an increased number of small, mature-appearing lymphocytes and several smudge cells, whereas leucoerythroblastic reaction was also noted, as is shown in Figure 1a. A flow cytometry (FC) test revealed the presence of a pathological population of B-lymphocytes positive for CD5, CD23, CD43, and CD200, with intermediate CD20 expression and weak CD79b and surface light chains expression, findings consistent with B-CLL.

Due to remarkably high levels of ALP and LDH, abnormal coagulation studies, and leucoerythroblastic reaction, which are uncommon findings in CLL patients, a search for other etiologic factors was followed. Upon investigation, strikingly high levels of prostate-specific antigen (PSA, 3,429 ng/mL; ULN 6.2 ng/mL) were found. Of note, the patient reported normal PSA levels eight months ago. A prostate ultrasound yielded no findings. No prostate biopsy was performed, given the deranged coagulation studies.

The patient was administered fresh frozen plasma and tranexamic acid, and a BM biopsy and aspiration were performed. Although aspirate was hard to obtain, the BM smear indicated the presence of clusters of non-hematopoietic cells, as shown in Figures 1b-1c. Histologic evaluation (Figures 1d-1h) revealed infiltration of the BM by a poorly differentiated prostate cancer and the presence of extensive necrosis. Immunohistochemical staining was positive for ΝΚΧ3.1, ΑΕ1/ΑΕ3 and CK19 (dim) and negative for CK20, TTF-1, CK7, CDX2, GATA-3 and napsin A1. Notably, as is seen in Figure 1h, infiltration of BM by CLL cells was not observed.

**Figure 2:**
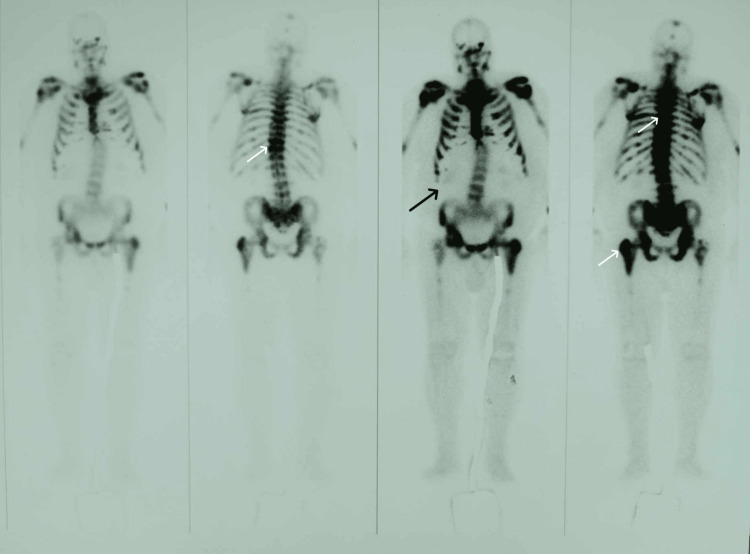
Tc-99m diphosphonate bone scan of the patient demonstrating a superscan pattern indicative of bone metastases White arrows indicate sites of increased skeletal radioisotope uptake, while the black arrow points to diminished renal uptake. This symmetrically intense and diffuse skeleton radioisotope uptake, in combination with the diminished renal and soft tissue uptake (superscan appearance), confirms the presence of extensive bone metastases.

Further imaging with whole-body computed tomography (CT) demonstrated multiple sclerotic lesions with ill-defined margins, involving the sternum, vertebral bodies, and femurs, likely reflecting secondary bone involvement, while a bone scintigraphy revealed a superscan appearance, as is depicted in Figure 2, confirming the occurrence of extensive bone metastases.

The patient was started on GnRH analogues (Degarelix) and denosumab as a palliative treatment for his prostate cancer, whereas no treatment intervention for CLL was required since anemia and thrombocytopenia were attributable to metastatic prostate cancer. The patient achieved a significant reduction in PSA and ALP levels along with a marked improvement in hemoglobin levels and platelet count. Six months after treatment, the patient had normal PSA and ALP levels, and the hematologic parameters were within normal range.

## Discussion

The emergence of chemoimmunotherapy and targeted agents has significantly increased overall survival (OS) rates in CLL. However, this has also led to a higher incidence of second primary malignancies during the course of the disease. The elevated risk of developing SMs in CLL patients has long been acknowledged [5]. The inherent abnormal cellular and humoral-mediated immune responses, stemming from quantitative and qualitative defects in immune-mediator cells in CLL, lead to compromised immune surveillance of tumors. It is still uncertain to what extent this heightened risk of SMs is due to CLL itself and associated chronic immune suppression, or to the various antineoplastic treatments used against CLL. Multiple studies have indicated that patients with CLL face an increased risk of developing skin cancer, SOTs, and HMs [5-12]. The study populations for these research studies include patients who had not undergone therapy, those who had received chemoimmunotherapy, and patients treated with novel targeted agents. Data from the largest studies examining cancer risk in CLL patients are summarized in Table 2.

**Table 2:** Cancer risk in patients with chronic lymphocytic leukemia SOTs: solid organ tumors; HMs: hematological malignancies; SM: secondary malignancy; FCR: fludarabine, cyclophosphamide, rituximab; BTKi: Bruton’s tyrosine kinase inhibitor

Study	Number of Patients	Follow-Up	Malignancies	Relative Risk	
Manusow and Weinerman, 1975 [5]	102	19 years	SOTs and skin cancers	3-fold for all cancers, 8-fold for skin cancers	
Hisada et al. 2001 [6]	16,367	5.2 years	SOTs and skin cancers	Observed/expected ratio 1.20	
Benjamini et al. 2015 [7]	243	4.4 years	SOTs and HMs	2.38 after FCR therapy	
Ishdorj et al. 2019 [8]	587	6.65 years	SOTs and skin cancers	4-fold for skin cancers	
Eversman et al. 2020 [9]	265	10.5 years	SOTs, skin cancers, and HMs	30.9% SM, cumulative incidence of SMs not different among treatment groups, cumulative incidence of cutaneous malignancies higher among patients treated with targeted agents	
Bond et al. 2020 [10]	691 on BTKi	44.5 months	SOTs, skin cancers, HMs, Richter’s Syndrome	2.2 for second primary malignancies	
Shen et al. 2021 [11]	517	11 years	Skin cancers, SOTs, HMs, and Richter’s syndrome	Standardized Incidence Ratio 3.81	
Van der Straten et al. 2023 [12]	24,815	6.2 years	SOTs and HMs	Standardized Incidence Ratio 1.63	

Data regarding the time from the diagnosis of CLL until the SM diagnosis is generally scarce. A study has previously demonstrated a median time of four years [9]. Our patient had a concurrent diagnosis of CLL and prostate cancer, thus indicating that SMs may occur even in early stages or at diagnosis of CLL. A diagnosis of SM in CLL patients may be troublesome, since its presenting features may be masked by CLL symptoms or may be considered to be CLL-associated. Our patient had profound anemia and thrombocytopenia, which could have been attributed to CLL and led to treatment initiation. However, the patient also had exceptionally high levels of ALP and LDH, deranged coagulation studies, as well as leucoerythroblastic reaction in his blood smear, which are unexpected findings in CLL, and ultimately led to an advanced-stage prostate cancer diagnosis with BM metastases, thus explaining the patient’s cytopenias. Hence, physicians should stay vigilant for signs and symptoms that are unusual in CLL or are suggestive of malignancy, since their prompt recognition may not only result in a SM diagnosis but may also have therapeutic implications for CLL. Notably, leucoerythroblastic reaction, markedly elevated ALP, and coagulation abnormalities, particularly in male patients, should be regarded as potential red flags for metastatic prostate cancer that warrant prompt diagnostic evaluation.

CLL patients with cancer usually display a rather aggressive clinical course and worse survival rates than patients with cancer alone [4, 6]. The National Comprehensive Cancer Network (NCCN) recommends avoidance of risk factors along with annual dermatologic evaluations for melanoma and non-melanoma skin cancer screening [13]. The NCCN also highlights the importance of strict adherence to established screening guidelines for other SOTs [13]. Our patient was diagnosed with advanced-stage prostate cancer, with metastatic disease involving the bones and the BM, although he had a negative screening test for prostate cancer eight months earlier. This rapid transition from a negative screening test to disseminated disease might reflect the aggressive nature of SM in CLL patients and also underscores potential limitations of applying standard screening intervals in this high-risk patient group. Hence, it may be reasonable for CLL patients to undergo screening tests for cancers more frequently than the general population. For instance, for prostate cancer screening, male CLL patients might benefit from undergoing PSA testing twice a year.

A large study has previously reported that patients with pre-existing CLL and cancer had an inferior OS, as compared to patients without CLL at the diagnosis of breast, colorectal, and prostate cancer, whereas the inferior OS remained when the CLL-associated deaths were excluded from the analysis [6]. It remains unclear why CLL patients have unfavorable cancer-specific outcomes. It could be related to either more aggressive biological characteristics of SM in CLL patients or less effective immune surveillance/control. Individuals with CLL have impaired immune responses, which may create an environment more permissive to local invasion and/or metastasis. It is also possible that they receive less aggressive treatment, considering their underlying immunosuppression and cytopenias. Notably, in our patient, a complete absence of BM infiltration by CLL cells was observed, potentially reflecting the aggressiveness of SOTs in this setting and their capability to dominate the BM environment. Nevertheless, this finding warrants further research, particularly regarding the state of BM in cases of dual pathology involving CLL.

## Conclusions

In conclusion, unusual clinical or laboratory findings in patients with CLL, even in early stages, should prompt the diagnosis of a SM. Considering their aggressive clinical course and their association with worse survival, a strategy of more frequent cancer screening, such as biannual PSA tests, may allow for earlier identification of SMs. This case highlights the need for further research to determine screening intervals for SMs in this high-risk population. Establishing such guidelines could optimize early detection and improve outcomes in CLL patients.
